# Synthesis of Copper and Copper Oxide Nanomaterials by Pulsed Electric Field in Water with Various Electrical Conductivities

**DOI:** 10.3390/nano10071347

**Published:** 2020-07-10

**Authors:** Ahmad Hamdan, Xavier Glad, Min Suk Cha

**Affiliations:** 1 Groupe de Physique des Plasmas, Département de Physique, Université de Montréal, 1375 Avenue Thérèse-Lavoie-Roux, Montréal, QC H2V 0B3, Canada; xavierglad@gmail.com; 2 Clean Combustion Research Center (CCRC), Physical Science and Engineering Division (PSE), King Abdullah University of Science and Technology (KAUST), Thuwal 23955, Saudi Arabia; MinSuk.Cha@kaust.edu.sa

**Keywords:** copper oxide, nanoparticles, nanowires, electric field, electrolysis

## Abstract

Nanomaterial synthesis is a hot research subject that has been extensively studied in the last two decades. Recently, plasmas in liquid systems have been proposed as an efficient means of synthesizing various types of nanomaterials. The formation processes implicate many physical and chemical phenomena that take place at the electrode surface, as well as in the plasma volume, which renders it difficult to fully understand the underlying mechanisms. In this study, we assess the effect of electric field on nanomaterial synthesis in a system composed of two copper electrodes immersed in water, in the absence of an electrical discharge. The obtained results indicate that various nanostructures, including copper nanoparticles, copper oxide nanowires, and/or hollow nanoparticles, may be produced, depending on the electrical conductivity of the solution (adjusted by adding highly diluted HCl to deionized water). The materials synthesized herein are collected and characterized, and a formation mechanism is proposed. Overall, our results provide insight into the physical and chemical phenomena underlying nanomaterial synthesis in plasmas in liquid.

## 1. Introduction

Nowadays, nanotechnology is widely used in numerous applications, including energy storage and conversion, photonics, and biomedical applications [1,2]. However, despite the advantages and wide applicability of nanomaterials, their synthesis routes are not been fully understood [3]. Currently, research efforts are focused on developing ecological means of nanomaterial synthesis that are cost effective, highly efficient, and require minimal use of chemicals [4].

The synthesis methods developed in the last century may be categorized as top-down or bottom-up methods. The former methods implicate the breaking down of macroscopic material to nanoscale level (miniaturization), and they include mechanical ball milling [5] and lithography [6]. Meanwhile, the latter methods, such as molecular self-assembly [7] and atomic layer deposition [8], rely on building nanoscale materials from atoms and molecules. In general, nanomaterial synthesis methods make use of either physics- or chemistry-based technologies. However, hybrid methods based on plasma techniques have been recently proposed [9,10,11]. In particular, reactive plasmas that are not in thermodynamic equilibrium show great potential for use in applications of nanomaterial synthesis [12,13,14].

Recently, plasmas in or in contact with a liquid medium have been proposed as an efficient means to produce nanomaterials [15,16,17,18]. Plasmas in contact with liquids implicate reactions between plasma electrons and the metal ions contained in solution, and they have shown great success in the synthesis of various nanomaterials [17,18]. Meanwhile, plasmas in liquids produce nanomaterials by reacting the eroded electrode particles with plasma species [19,20]. The reactions also depend on the liquid composition. Both types of plasmas are known to be ecofriendly and to produce high yields of novel nanomaterials due to their unique characteristics of liquid confinement, high electron density, high pressure, and reduced lifetime [21]. 

Plasmas in liquid are usually generated by pulsed electrical discharges via the application of an intense and abrupt electric field (>100 kV/cm). Typically, the creation of a spark discharge in deionized water between two electrodes requires an interelectrode gap of about 100 µm, a voltage of around 10 kV, and a pulse-width of ~100–500 ns [19,20,21]. It should be noted that the electrode surface experiencing the high electric field is much larger than the one exposed to the plasma discharge (discharges in liquid lead to the formation of micrometric impacts (diameter of tens of micrometers) at the electrode surface [19]). The influence of the electrical field (up to 900 V/m) on nanomaterial synthesis (induced by laser ablation) was found to be significant [13]. Specifically, the authors have shown that the average nanoparticle size decreases with increasing electric field strength.

Previously, we had investigated the formation of Cu-based nanomaterials by electrical discharges in water with various electrical conductivity (adjusted by adding HCl to deionized water at very low concentration) [22]. Depending on the initial conductivity value, various nanostructures were observed, including nanoflakes of Cu_2_O, dendrites with high Cu content, and unordered micrometric aggregates with a mixed Cu/Cu_2_O content. Although the synthesis of nanoparticles was achieved by sustaining discharges in the solution, the high electric field may contribute to the synthesis process. In this study, our aim is to evaluate the sole effect of electric fields on the synthesis of Cu-based nanomaterials in water. Based on the obtained data, a synthesis mechanism is proposed. 

## 2. Experimental Setup

**Figure 1:**
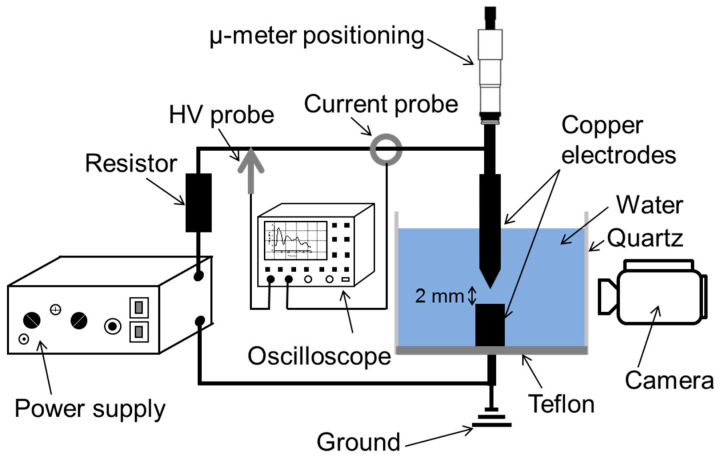
Schematic of the experimental setup used to synthesize Cu-based nanomaterials by applying pulsed voltage on Cu electrodes immersed in water.

As shown in Figure 1, the experimental setup consisted of a quartz cylinder (inner- and outer-diameters of 5 and 7 cm, respectively) mounted on a Teflon base. Two cylindrical Cu electrodes (2 mm diameter, 99.95% purity; Goodfellow) were vertically mounted in the liquid contained in the cylinder. The upper electrode (anode) was movable and had a polished needle-like tip with an apex angle of ~35°, whereas the lower one was grounded and had a flat tip. A high-voltage generator (Cober electronics Inc., model 606) was used to generate electrical pulses at a repetition rate of 13 Hz. The interelectrode distance was maintained at 2 mm to avoid discharges. Voltage (V) and current (I) were measured using a high-voltage probe (Tektronix, model P6015a) and a wide band terminated current transformer (LILCO LTD, model 13w5000), respectively. The electrical characteristics were visualized and acquired using a Tektronix DPO 5204B oscilloscope, whereas the electrode region was monitored using a camera video (Panasonic, AG HMC70). 

Before each experiment, every part of the apparatus was thoroughly cleaned with acetone, methanol, and finally, deionized water. The clean quartz cell was then filled with 50 mL of water whose conductivity (σ) and acidity (pH) were measured before and after each run using a conductivity meter (Cole-Palmer Instrument) and a pH meter (VWR SB20), respectively. To assess the effect of initial water electrical conductivity (σ_0_) on the efficiency of nanomaterial production, aqueous samples with σ_0_ = 2, 8, 32, and 64 µS/cm were tested. The initial electrical conductivity of water was adjusted by adding highly diluted HCl solution to deionized water (σ_0_ = 2 µS/cm). The HCl concentrations of all prepared water samples are listed in Table 1.

After each run, some of the synthesized nanomaterials were retrieved and laid on a 304-grade stainless steel (SS) substrate and a nickel mesh 300 TEM (transmission electron microscopy) lacey-carbon grid. To estimate the consumption rate, the pin electrode was imaged before and after each experiment using an optical microscope (OMAX, M83MPTR). The nanoparticles collected on the SS substrate were analyzed by a scanning electron microscope (SEM; JEOL, JSM6700F) in secondary emission mode (at 3 kV) and by energy dispersive X-ray spectroscopy (EDS, at 5 kV). Meanwhile, those collected on the Ni grid were analyzed by a transmission electron microscope (TEM; JEOL, JEM-2100F) operated at 200 kV in bright field, selected area electron diffraction (SAED), and EDS.

## 3. Results

### 3.1. Material Synthesis

**Figure 2:**
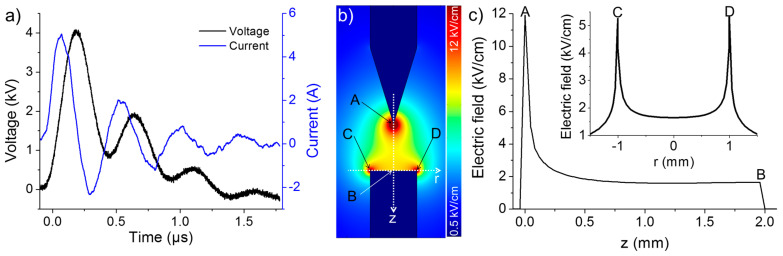
(**a**) Typical voltage-current characteristics for an applied voltage of 4 kV at σ_0_ = 2 µS/cm. (**b**) 2D-simulation of the electric field distribution. (**c**) Axial and radial profiles of electric field intensity along the electrode axis and the cathode surface derived from the simulation shown in (**b**).

Based on the results of preliminary tests, the interelectrode gap distance was fixed at 2 mm to avoid discharge for a given electrical circuit with a pulsed high voltage (4 kV, 13 Hz). The waveforms of voltage and current showed no appreciable difference for various σ_0_, which might be due to the relatively low concentration of HCl (10^−7^ M) added. As shown in Figure 2a, the waveforms acquired at σ_0_ = 2 µS/cm exhibit principal and secondary voltage peaks of ~4 and ~2 kV, respectively. Both peaks are approximately 250 ns wide. Meanwhile, the measured current, known as the displacement current, has principal and secondary peaks of ~5 and ~2 A, respectively.

In the absence of discharge, the synthesis of Cu-based nanomaterials was mainly induced by the action of the pulsed electric field and displacement current on the electrodes. Therefore, the spatial distribution of the electric field should play a primordial role in the process. To evaluate this distribution, 2D simulations resolving Laplace’s equation (Δ*V* = 0, where *V* is the electrical potential) were conducted using the COMSOL Multiphysics Software. Figure 2b illustrates the distribution of the electric field in the deionized water (relative permittivity of 80) at an applied voltage of 4 kV. Meanwhile, the axial and radial profiles of electric field intensity along the electrode axis and the cathode surface, respectively, are shown in Figure 2c. Based on the simulated data, two regions of high electric field can be identified: the anode tip (~12 kV/cm) and the rim of the cathode surface (~5 kV/cm). As described hereafter, the interesting phenomena related to nanomaterial synthesis/accumulation occur in these two regions. It should be noted that the simulation conducted here was very simplified and did not consider the space charge in solution. A detailed description of the space charge dynamic in dielectric liquids can be found in [23].

**Figure 3:**
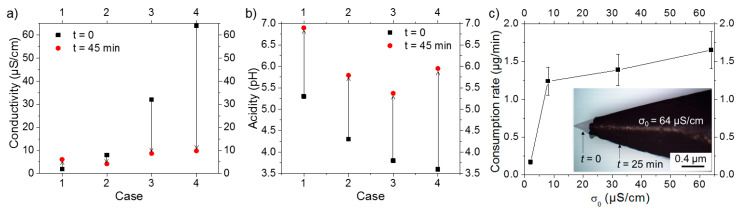
Variation of water (**a**) conductivity and (**b**) acidity before (at *t* = 0) and after (at *t* = 45 min) processing. (**c**) Effect of σ_0_ on the rate of matter consumption (the inset shows the anode tip at *t* = 0 and *t* = 25 min).

Water conductivity and acidity were measured before and after each experiment. As shown in Figure 3a, the generation of an electric field increased the conductivity of water, initially at 2 µS/cm (i.e., case 1), by ~5 µS/cm. However, for σ_0_ = 8, 32, and 64 µS/cm (i.e., cases 2, 3, and 4, respectively), a decrease in water conductivity was observed. For water acidity, Figure 3b shows that the pH values of σ_0_ = 2, 8, and 32 µS/cm solutions (i.e., cases 1, 2, and 3, respectively) increased by ~1.5, while that of σ_0_ = 64 µS/cm solution (i.e., case 4) rose by ~2.4. The amount of matter consumed from the anode under the effect of electric field was estimated based on the optical microscopy image of the anode tip (inset of Figure 3c). The effect of initial water conductivity on the consumption rate of matter is depicted in Figure 3c; note that the consumption rate is comparable to the nanomaterials production rate. The results indicate that the consumption rate increases sharply (from 0.2 to ~1.2 µg/min) upon increasing σ_0_ from 2 to 8 µS/cm. As σ_0_ further increased over 8 µS/cm, the consumption rate also increased, but at a slower rate. Here, because we considered the initial and final state of the electrode, only an average rate is provided. Although providing a temporal evolution of this rate is of importance, it is expected to decrease as a function of the processing time, because of the modification of the electrode tip as well as the decrease of water conductivity.

**Figure 4:**
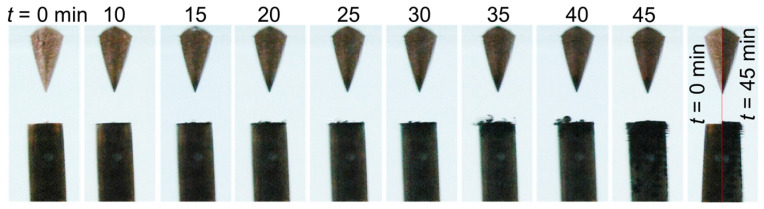
Temporal evolution of the liquid-electrode zone for case 1 (σ_0_ = 2 µS/cm).

The interelectrode gap and its vicinity were continuously monitored using a video camera. Figure 4 presents a series of images recorded between 0 and 45 min for case 1 (σ_0_ = 2 µS/cm). These images show that, with time, the cathode electrode and the tip of the anode electrode turned black, and micrometric bubbles started to appear and grow on its surface (15–40 min). A comparison of two images recorded at *t* = 0 and 45 min shows a ~200 µm-thick layer of deposition at the cathode surface. Note that this added material had been solely formed due to the presence of a strong electric field. 

**Figure 5:**
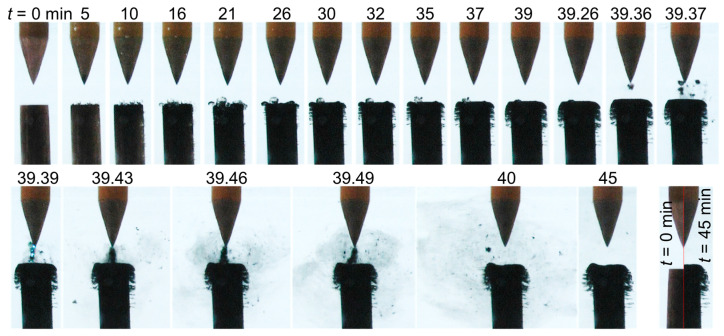
Temporal evolution of the liquid-electrode zone for case 2 (σ_0_ = 8 µS/cm).

As shown in Figure 5, case 2 (σ_0_ = 8 µS/cm) also presented bubbles lying on the cathode surface (5–35 min). At the same time, the cathode turned black, and brush-like filaments grew laterally along its radial circumference. The number and length of these filaments increased with time, and the cathode shape eventually appeared mushroom-like. 

Figure 5 also introduces a peculiar phenomenon which is described here with a smaller timescale. At *t* = 39.36 min, deposited materials started to detach from the cathode and oscillate in the gap between the two electrodes (supplementary film available on demand). The produced materials, which oscillated between the electrodes, seem to serve as guide space charges, resulting in the occurrence of an electrical discharge as observed at *t* = 39.39 min. It should be noted that, while it was also seen in other conditions, this brief spark discharge was only observed once during the entire experiment in these conditions. Shortly after this event, the materials formed a bridge between the electrodes, and the discharge was no longer observed. This strongly suggests that the synthesized solids forming the bridge were not conductive. Eventually, the bridging materials diffused in the liquid, but the number and length of the filaments on the radial circumference continued to increase. After 30 and 45 min of processing, the thickness of the deposited layer formed at the top of the cathode attained ~180 and ~400 µm, respectively. The latter thickness is highlighted in the last picture (bottom right) in Figure 5.

**Figure 6:**
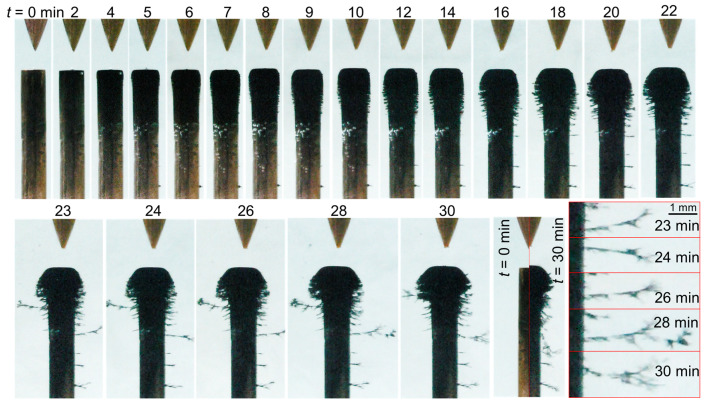
Temporal evolution of the liquid-electrode zone for case 4 (σ_0_ = 64 µS/cm).

The phenomenological behavior of the material formation in case 4 (σ_0_ = 64 µS/cm, Figure 6) was similar to that of case 2 (Figure 5); however, in case 4, the quantity of material deposited at the cathode surface was far greater. The thickness of the deposited layer formed after 30 min of processing was ~250 µm, and the length of the filaments growing on the radial circumference of the cathode was greater than 3 mm. The grown filaments had a fractal/dendritic-like structure (see the bottom-right picture in Figure 6).

**Figure 7:**
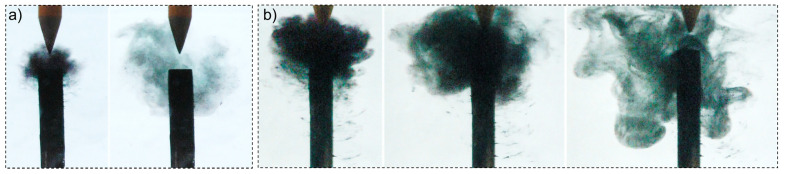
Release of materials from the cathode surface after switching off the power supply: (**a**) case 2 (σ_0_ = 8 µS/cm) and (**b**) case 4 (σ_0_ = 64 µS/cm).

It is worth noting that once the high voltage was switched off, significant amounts of the materials, previously collected at the cathode, were ejected and slowly diffused within the solution towards the bottom of the chamber. Such ejection implies that the synthesized materials were electrostatically bound to the cathode (Figure 7).

### 3.2. Material Characterization

The TEM analyses shown hereafter were performed on as synthesized nanomaterials after evaporating the solution on TEM grids. Also, we observed that the produced nanomaterials were stable in solution, at least tens of days after synthesis. Such stability of nanomaterials was not investigated after being exposed to ambient air or to other environment (e.g., annealing).

**Figure 8:**
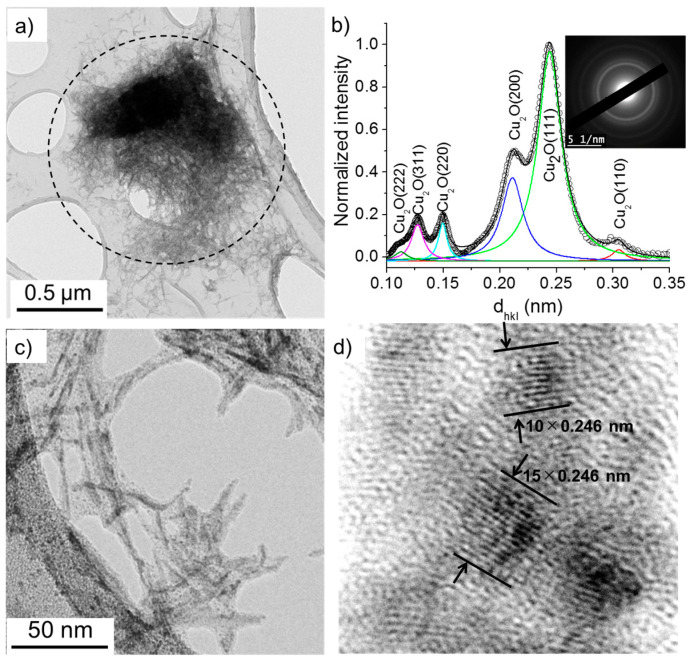
(**a**) TEM images of nanowires synthesized in deionized water (σ_0_ = 2 µS/cm) and (**b**) the corresponding selected area electron diffraction (SAED) pattern (performed on the encircled zone in (**a**)) radially integrated spectrum. (**c**) and (**d**) show the HR-TEM images of nanowires.

**Figure 9:**
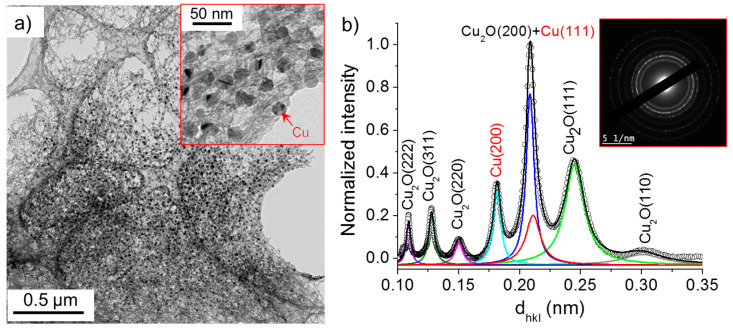
(**a**) TEM images of nanowires and nanoparticles synthesized in deionized water (σ_0_ = 2 µS/cm) and (**b**) the corresponding SAED pattern (performed on the insert in (**a**)) radially integrated spectrum.

The TEM images presented in Figure 8 and Figure 9 show that two kinds of nanomaterials may be produced in the deionized water (case 1) by the electric field: (i) nanowires and (ii) nanoparticles. The nanowires were characterized by their length and width ranging between 50 and 100 nm and 3 and 10 nm, respectively. Their SAED patterns show concentric circles with radii corresponding to specific interplanar distances, *d_hkl_*, which allow the determination of their crystalline structure. Herein, we used a previously developed method [22,24] to transform the SAED pattern into a radially integrated spectrum where each peak corresponds to the sum of the different diffraction spot at fixed *d_hkl_* values (i.e., fixed radius from the non-diffracted beam, in the reciprocal space). The peaks identified in Figure 8b thus suggest that the nanowires are composed of Cu_2_O crystals (*d*_(110)_ = 3.012 Å, *d*_(111)_ = 2.464 Å, *d*_(200)_ = 2.136 Å, *d*_(220)_ = 1.510 Å, *d*_(311)_ = 1.287 Å, and *d*_(222)_ = 1.232 Å). The high-resolution-TEM image (Figure 8d) further confirms that the nanowires are made of 2–5 nm Cu_2_O nanoparticles.

Some agglomerates found within the collected materials showed nanostructures with single nanoparticles in the size range of 5–20 nm (Figure 9a). The SAED pattern and resulting radially integrated spectrum of these agglomerates presented interplanar distances corresponding to Cu_2_O crystals as well as Cu (*d*_(111)_ = 2.09 Å and *d*_(200)_ = 1.81 Å) (Figure 9b). This indicates that the nanoparticles were not oxidized (if oxidized, a shift in *d* towards smaller values is usually observed [22,25]). Knowing that Cu can be easily oxidized in water or upon exposure to ambient air, one may assume that the synthesized Cu nanoparticles were embedded in a matrix of Cu_2_O nanowires, which may protect Cu particles from oxidation.

**Figure 10:**
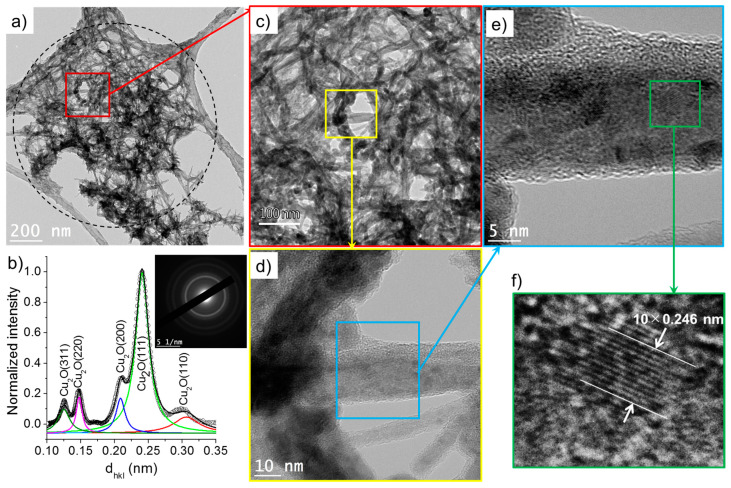
(**a**) TEM images of nanowires synthesized in the case 2 (σ_0_ = 8 µS/cm) and (**b**) the corresponding SAED pattern (performed on the encircled zone in (**a**)) radially integrated spectrum. (**c**–**f**) show the HR-TEM images of nanowires.

**Figure 11:**
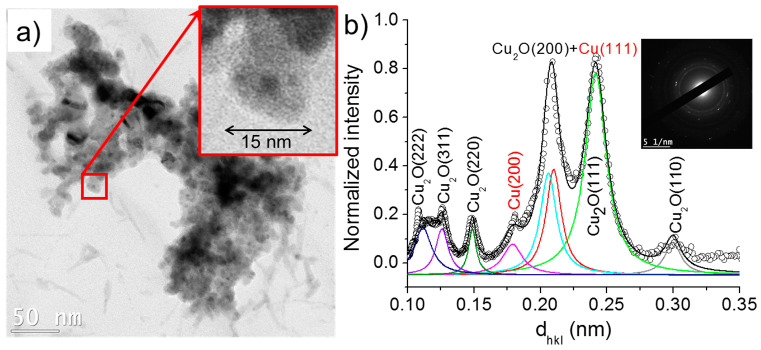
(**a**) TEM images of nanoparticles synthesized the case 2 (σ_0_ = 8 µS/cm) and (**b**) the corresponding SAED pattern (performed on (**a**)) radially integrated spectrum.

Figure 10 and Figure 11 show that the nanowires and nanoparticles were also found in the case 2. Based on the acquired SAED pattern and spectrum, the nanowires were mainly composed of Cu_2_O. Their lengths and widths ranged from 50 to 100 nm and 3 to 10 nm, respectively, similarly to the nanowires synthesized in deionized water (case 1). The high-resolution TEM images depicted in Figure 10d–f confirm the presence of Cu_2_O crystals (3–5 nm) within the nanowires. 

As shown in Figure 11, the nanoparticles formed in the case 2 (σ_0_ = 8 µS/cm) have a size distribution of 5–20 nm and are composed of Cu_2_O and Cu. The inset of Figure 11a, which depicts a typical nanoparticle, demonstrates a higher contrast (darker zone) at its center. In TEM imaging, although the contrast depends on Z (the atomic number), the relative distribution of atoms (Cu and O in our case) in a plane also led to the reduction in contrast, indicating thus an oxidized phase [24]. This hints that the nanoparticles hereby observed have a core–shell structure (Cu-rich core and Cu_2_O-rich shell).

**Figure 12:**
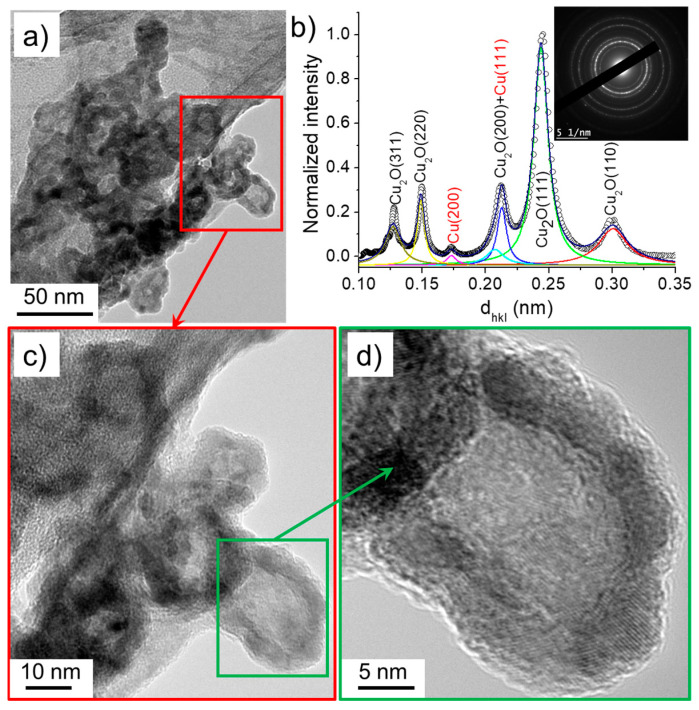
(**a**) TEM images of nanoparticles synthesized in the case 3 (σ_0_ = 32 µS/cm) and (**b**) the corresponding SAED pattern (performed on (**a**)) radially integrated spectrum. (**c**) and (**d**) show the HR-TEM images of nanoparticles.

The nanowires identified in case 3 (σ_0_ = 32 µS/cm) had a similar size distribution to those synthesized in solutions of lower conductivities (cases 1 and 2) and were composed of Cu_2_O. Meanwhile, the size of the nanoparticles varied between 20 and 30 nm, which was slightly larger than those obtained under lower conductivity conditions. Moreover, unlike the previously discussed cases, the nanoparticles featured in case 3 were characterized by higher contrast shells than those found in cases 1 and 2 (Figure 12). This suggests a hollow-like structure, similarly to the ones reported by Nakamura et al. [26]. The nanoparticles’ SAED pattern and its radially integrated spectrum indicate that they were mainly composed of Cu_2_O. Note that the nanomaterials formed with the solution of highest conductivity (case 4, σ_0_ = 64 µS/cm) were very similar to those identified in the case 3: hollow nanoparticles and nanowires of Cu_2_O (not shown here). 

## 4. Discussion

The identification of two types of nanostructures synthesized under the effect of the electric field indicates that at least two mechanisms take place during the process. One mechanism forms the nanoparticles composed of Cu (σ_0_ = 2 µS/cm), Cu and Cu_2_O (σ_0_ = 8 µS/cm), or Cu_2_O (σ_0_ = 32 and 64 µS/cm), while the other should be responsible for the Cu_2_O-based nanowires (found under all investigated conductivity conditions). It is worth noting that in each conductivity condition major particles are shown. However, other minor particles could be present. For example, at higher conductivity (σ_0_ = 32 µS/cm), it was possible to find few particles of Cu (similar to those found at σ_0_ = 2 µS/cm or Cu-Cu_2_O core-shell (similar to that found at σ_0_ = 8 µS/cm), in addition to the majority of hollow Cu_2_O nanoparticles. Therefore, we believe that the transition between particle shape as a function of solution conductivity is smooth and depends on the local concentration of negative species that can accumulate at the particle surface enhancing, thus, the Kirkendall effect occurring at the Cu/Cu_2_O interface. 

The synthesis mechanisms are derived from that of the standard electrolysis processes. At the anode, the following oxidation reactions are dominant [27]:

2Cu → 2Cu^2+^ + 4e^−^ (1)

2H_2_O → O_2_(O–O)_SA_ + 4H^+^ + 4e^−^ (2)

where SA stands for surface adsorbed species. Whereas at the cathode, the reduction reactions are:

4H^+^ + 4e^−^ → 2H_2_ (3)

2Cu^2+^ + 4e^−^ → 2Cu (4)

Therefore, Cu^2+^ and oxygen adsorbed species are expected to be identified near the anode. That there are missing bubbles at the anode, as shown by the video images recorded herein, supports the fact the produced oxygen species are adsorbed at the surface. The adsorbed oxygen then reacts with Cu^2+^ via Equation (5), leading to the formation of Cu_2_O nanomaterials in the anode area.

2Cu^2+^ + (O–O)_SA_ + 4e^−^ → Cu_2_O + O_SA_ (5)

The Cu_2_O products eventually accumulate at the anode surface, resulting in the growth of Cu_2_O nanowires that are oriented perpendicularly relative to the electrode surface. This mechanism of Cu_2_O nanowire growth has been previously proposed in electrolysis processes [27]. Their growth as nanowires and not as thin film could be related to inhomogeneity of the electrode surface, as protrusions may exist, where the growth is initiated. The detection of nanowires in the solution and their accumulation at the cathode (as the video images showed) suggests that, at some point, these products detach from the anode. This phenomenon has also been observed in electrolysis processes [27]. Also, in anodization of copper induced by plasma in contact with deionized water, Kim et al. [28] have observed the dissolution of the oxidized layer (grown at the copper anode surface) in water, and very similar nanowires are identified in solution. Once detached from the anode, the nanowires are polarized by the electric field and are transported to the cathode region where they accumulate at the cathode surface. Considering that similar nanowire structures and compositions were recorded under varying conditions of initial water conductivity, it may be assumed that the mechanism of wire growth is less sensitive to the electrical conductivity, at least within the tested range. However, it should be noted that, as a result of adding HCl (increased conductivity), the yield of nanowire production is significantly enhanced. In fact, it has been previously reported [29] that for chloride concentrations less than 10^−4^ M (which is the case here), a film of CuCl is grown at the anode. Because this film gets oxidized via reactions (Equations (6) and (7)), an enhancement of the yield of nanowire synthesis is thus expected.

2CuCl + H_2_O → Cu_2_O + 2H^+^ + 2Cl^−^ (6)

2CuCl + 2OH^−^ → Cu_2_O + H_2_O + 2Cl^−^ (7)

On the other hand, under the action of the electric field, some Cu^2+^ species migrate to the cathode where they recombine with electrons to form Cu (Equation (4)) and therefore Cu nanoparticles. The size of the nanoparticles does not vary significantly under various water conductivities. However, their structure and chemical composition are substantially affected by conductivity. At σ_0_ = 2 µS/cm, quasi-spherical Cu nanoparticles are mainly produced, whereas at σ_0_ = 32 and 64 µS/cm, the particles are primarily composed of hollow Cu_2_O structures. The synthesis of hollow metal oxide nanocrystals, including copper oxide, has been extensively studied and usually involves two distinct processes: surface oxidation and vacancy coalescence induced by outward diffusion of the metal atoms. Metals (e.g., Fe, Zn, Al, and Cu) undergo surface oxidation (thickness of several nanometers) in ambient air at room temperature. The formation of hollow nanoparticles usually occurs in aqueous solutions at elevated temperature which enhances the outward diffusion of metal ions from the core to the shell. However, according to Cabrera and Mott [30] and Hung et al. [31], oxidation in a solution at room temperature can also lead to hollow structures under the action of a sufficient electric field. Indeed, the adsorption of oxygen atoms onto the oxide surface leads to electrons penetrating the oxide layer by tunnel effect. Because of the high diffusion rate of Cu in copper oxides, the process results in the transport of the metal ions from the core to the shell (Kirkendall effect occurring at the Cu/Cu_2_O interface). In this study, hollow nanoparticles could only be produced under high conductivity conditions wherein Cl species are expected to be adsorbed at the surface of the synthesized nanoparticles (EDX spectra on these particles have revealed the presence of traces of Cl, not shown here), thereby enhancing the electric field and promoting the migration of core ions. Note that, globally, the temperature of the solution did not change during the process and remains at room temperature. However, a local increase in the regions where the electric field is high (e.g., close to the tip of the pin electrode) could be expected.

As mentioned earlier, the aim of this study is to highlight the role of electric field in the synthesis process during electrical discharges. In discharges, under similar conditions, three major families of nanostructures were identified: unordered micrometric agglomerates (UMAs), nanoflakes, and dendrites (for more details, see [22]). The UMAs consist of Cu and Cu_2_O nanoparticles and are detected at all investigated conductivity conditions (2, 4, 8, 16, 32, and 64 μS/cm). The increase of σ_0_ was accompanied by a rise in Cu_2_O content. The nanoflakes were detected at σ_0_ ≥ 16 μS/cm, and they consist of larger submicronic Cu_2_O crystals joined together in a single 2D plane by grain boundaries. As for the dendrites, they are predominantly found at low σ_0_ and are primarily comprised of Cu. Table 2 provides a comparison of the nanomaterials produced in both processes, electric-field-only and discharges. Nanowires are never observed during discharges but only under electric-field-only conditions. This observation indicates that, if grown at the electrode surface, the nanowires are continuously destroyed by the repetitive discharges and injected into the medium as nanoparticles. As for the nanoflakes, their absence under electric-field-only conditions is to be expected. Indeed, they are supposed to be synthesized by the removal of an oxidized film at the electrode surface due to the discharge-induced shockwave [22]. In conditions enabling discharges, UMA and dendrites were observed, but not in electric field-only conditions. This fact can also be explained by the ejection of Cu particles, under the action of the discharge, and their assembly under high electric field (this field can be linked to the pulse period preceding the breakdown, failed discharges, and/or to the space charge during discharge) conditions owing to the dielectrophoresis mechanism. As for the hollow nanoparticles, their synthesis in electric-field-only and at high conductivity could be related to a relatively slow mechanism (Kirkendall effect) that is assumed not to be dominant during discharges.

## 5. Conclusions

In this study, we show that a pulsed electric field between two copper electrodes immersed in water leads to the synthesis of Cu-based nanomaterials. Depending on electrical conductivity of water (adjusted between 2 and 64 µS/cm by adding highly diluted HCl), different kinds of nanomaterials may be produced. At low conductivity (2 µS/cm), nanowires of Cu_2_O and nanoparticles of Cu are synthesized. Meanwhile, nanowires and hollow nanoparticles of Cu_2_O are produced at high conductivity (32 and 64 µS/cm). The proposed mechanism suggests that the nanowires grow at the anode, then, detach and accumulate at the cathode surface, as observed by video camera. On the other hand, the Cu nanoparticles synthesized in low conductivity conditions appear to be growing at the cathode. These nanoparticles transform to hollow Cu_2_O nanoparticles as water conductivity increases which may be explained by the Kirkendall effect occurring at the Cu/Cu_2_O interface. The results reported herein may be used to develop and enhance nanomaterial synthesis applications that make use of in-liquid discharges, especially in the presence of a relatively high electric field intensity.

## Figures and Tables

**Table 1:** Aqueous solution initial conductivities (σ_0_) and the corresponding HCl concentrations.

Case	1	2	3	4
**σ_0_ (µS/cm)**	2	8	32	64
**[HCl] (10^−7^ M)**	0	1	4	8

**Table 2:** Comparison of the main nanomaterials produced by electric field and by discharges under various conditions of water conductivity.

σ_0_ (µS/cm)	Products	
	With Electric Field	With Discharges [22]
**2**	Nanowires; Cu nanoparticles	unordered micrometric agglomerates of Cu/Cu_2_O; Cu dendrites
**8**	Nanowires; Cu/Cu_2_O nanoparticles	
**32**	Nanowires; hollow Cu_2_O nanoparticles	unordered micrometric agglomerates of Cu/Cu_2_O; Cu_2_O nanoflakes
**64**		
